# METTL14 Regulates Intestine Cellular Senescence through m^6^A Modification of Lamin B Receptor

**DOI:** 10.1155/2022/9096436

**Published:** 2022-12-19

**Authors:** Zizhen Zhang, Meng Xue, Jingyu Chen, Zhuo Wang, Fangyu Ju, Jiaojiao Ni, Jiawei Sun, Haoyue Wu, Huimei Zheng, Ziwei Lou, Yawen Zhang, Xiaohang Yang, Shujie Chen, Yongmei Xi, Liangjing Wang

**Affiliations:** ^1^Department of Gastroenterology, The Second Affiliated Hospital of Zhejiang University School of Medicine, Zhejiang, China; ^2^Institute of Gastroenterology, Zhejiang University, Zhejiang, China; ^3^Research Center of Prevention and Treatment of Senescent Disease, Zhejiang University School of Medicine, Hangzhou, China; ^4^The Woman's Hospital and the Institute of Genetics, Zhejiang University School of Medicine, Zhejiang, China; ^5^Department of Gastroenterology, Sir Run Run Shaw Hospital, Zhejiang University School of Medicine, Zhejiang, China

## Abstract

N-6-Methyladenosine (m^6^A) modification is involved in multiple biological processes including aging. However, the regulation of m^6^A methyltransferase-like 14 (METTL14) in aging remains unclear. Here, we revealed that the level of m^6^A modification and the expression of METTL14 were particularly decreased in the intestine of aged mice as compared to young mice. Similar results were confirmed in *Drosophila melanogaster*. Knockdown of Mettl14 in *Drosophila* resulted in a short lifespan, associated disrupted intestinal integrity, and reduced climbing ability. In human CCD-18Co cells, knockdown of METTL14 accelerated cellular senescence, and the overexpression of METTL14 rescued senescent phenotypes. We also identified the lamin B receptor (LBR) as a target gene for METTL14-mediated m^6^A modification. Knockdown of METTL14 decreased m^6^A level of LBR, resulted in LBR mRNA instability, and thus induced cellular senescence. Our findings suggest that METTL14 plays an essential role in the m^6^A modification-dependent aging process via the regulation of LBR and provides a potential target for cellular senescence.

## 1. Introduction

Aging is a systematic biological process which causes physiological deficits leading to many age-associated disorders including neurodegenerative diseases, cancer, and cardiovascular disease [1–4]. Recent studies have implicated a variety of epigenetic changes that occurs during the aging process [5], including histone methylation, histone acetylation [6], DNA methylation [7], and RNA modification [8]. N-6-Methyladenosine (m6A) is the most prominent epigenetic modification in messenger RNAs (mRNAs) and noncoding RNA (ncRNA) [9]. It plays pivotal roles in biological processes such as stemness maintenance, differentiation, and cell senescence [10, 11].

The m^6^A modification in RNA is a reversible chemical modification. It is regulated by the methyltransferase complex consisting of m^6^A “writers” (including METTL3, METTL14, and WTAP), m^6^A “erasers” (including FTO and ALKBH5), and m^6^A “readers” (including YTHDC1, YTHDC2, YTHDF1, YTHDF2, and HNRNPC) [12–14]. The downstream functions of m6A are mediated by reader proteins that regulate mRNA processing [15]. IGF2BP2, IGF2BP3, and YTHDF1 are a class of m6A readers which promote the stability of mRNAs [16–18]. Besides, there are various other regulations including mRNA stability, translational regulations, pre-mRNA splicing, and RNA nuclear export [12].

Previous studies have shown that METTL3/14 is progressively declined in cells that have undergone replicative senescence [19]. Global m6A modification level is also notably reduced in prematurely senescent human mesenchymal stem cells (hMSCs) [20]. Further studies found that depletion of METTL3 led to m^6^A modified *MIS12* mRNA downregulation, thus accelerating hMSC senescence [20]. Global m^6^A modification mainly occurs in mRNAs, and a subset of methylated mRNAs are subsequently decreased in old cohorts compared to young cohorts [21].

Lamin B receptor (LBR) is an evolutionary conserved, multifunctional protein [22]. Previous studies have shown that knockdown of LBR facilitates cellular senescence in HeLa cells and induces cellular senescence in normal human diploid fibroblasts [23]. Lack of LBR caused chromatin structure changes and altered metabolic and inflammatory processes, resulting in physiological aging [24]. Knockdown of LBR could upregulate the expression of the senescence-associated secretory phenotype (SASP) factors such as IL-6 and IL-8 in HeLa cells [25]. Recent study indicated the reduction of LBR by miR-340-5p as a mechanism whereby WI-38 human diploid fibroblasts promote senescence by reducing chromatin stability [26]. Studies also demonstrated that cytoplasmic chromatin foments (CCF), a senescence-associated proinflammatory program, underscore the important and novel role for LBR in maintaining heterochromatin homeostasis and avoiding senescence [27].

The intestine is an organ which undergoes considerable degeneration corresponding to the aging process [28, 29], whilst remaining indispensable for food digestion, nutrient absorption, and waste removal [30]. Upon aging, the intestinal barrier function becomes weakened, and the ability of digestion and absorption undergoes a corresponding decline. *Drosophila* is a useful model organism for studies on human aging [31]. For example, both *Drosophila* models and human studies have shown similarly increased intestinal permeability with aging [32, 33]. However, whether m^6^A RNA modification plays a role in gut senescence has not yet been well elucidated.

In this study, using mice, *Drosophila*, and a colon fibroblast-CCD18-Co cell line as models, we explored the effects of m^6^A modification on the gut function and cellular senescence. The m^6^A modification level showed considerable decreased in 18-month mice gut tissues comparing with those of 3-month mice. Knockdown of METTL14 in CCD18-Co cells also showed characteristics of accelerated senescence. Correspondingly, flies with Mettl14 depletion had a shorter lifespan and disrupted intestinal integrity. We concluded that METTL14 exerts a regulatory function via m^6^A modification in LBR RNA during aging that is highly correlated to cellular senescence.

## 2. Materials and Methods

### 2.1. Cell Culture and DOX-Induced Senescent Cells

The colon fibroblast cell line CCD-18Co was purchased from American Type Culture Collection (ATCC, Manassas, VA, USA). The cell line was cultured in Eagle's Minimum Essential Medium (EMEM; Genom, Hangzhou, China) supplemented with 10% fetal bovine serum (FBS; Sijiqing, Hangzhou, China) and 1% penicillin and streptomycin. The cell line was cultured in a monolayer culture in a humidified atmosphere containing 5% CO_2_ at 37°C. For senescence induction, cells were plated at a density of 200,000 per well of a six-well plate. For induction of senescence by doxorubicin (DOX; Sigma-Aldrich, St. Louis, MO, USA), CCD-18Co cells were cultured in the presence of 300 nM doxorubicin in complete culture media for 72 h. CCD18-Co cells were cultured to a confluent state and counted. Population doubling level (PDL) during each passage was calculated by the equation *A* = 3.32 (logN2 − logN1) + *X* (*A*, added population doubling level; N2, collected cell number at the end of each passage; and N1, cell number at the beginning of each passage). Cells were passaged until they did not show obvious proliferation (usually passage 18–20), which could be considered as high passage. Cells at higher passages and DOX-induced senescent cells were used for analysis.

### 2.2. Plasmid Construction and Lentivirus Preparation

For the METTL14 overexpression, the METTL14 coding region was inserted into a pLV-puro plasmid (GeneChem, Shanghai, China). For METTL14 or LBR knockdown, lentiviral vector (GV248) containing METTL14 or LBR was obtained from GeneChem (Shanghai, China). The shRNA sequences were listed in Table 1. HEK 293 T cells were transfected with abovementioned plasmids and the virus packing plasmids using PolyJet transfection reagent (SignaGen, Rockville, MD, USA). The virus-containing medium was harvested and filtered to remove cell debris after 48 h. CCD-18Co cell line was plated in six-well plates. 24 hours later, the cell lines were transduced with virus-containing medium to establish stably infected cell lines.

### 2.3. Reverse Transcription-Quantitative PCR (RT-qPCR)

For RT-qPCR assays, total RNA was extracted using the TRIzol^®^ Reagent (Invitrogen, Camarillo, CA, USA) following the manufacturer's protocols, and then, a total of 1 *μ*g RNA was converted into cDNA using PrimeScript™ RT Reagent Kit (Takara, Japan) according to the manufacturer's instructions. RT-qPCR was carried out using SYBR Premix Ex Taq (Takara, Japan) in a Light Cycler®480 Real-Time PCR System (Roche) using cDNA. The cDNA was then quantified using real-time PCR under the following conditions: denaturation at 95°C for 2 min, 40 cycles of 95°C for 15 s, and 60°C for 30 s. The primer sequence of related genes involved in the article is listed in Table 2. The 2^−*ΔΔ*Ct^ method was applied to calculate the relative mRNA expression. *β*-Actin served as internal normalized reference.

### 2.4. Western Blotting

Total proteins were extracted from CCD-18Co cells using a RIPA lysis buffer. The concentration of extracted proteins was measured using the BCA standard method (Beyotime Institute of Biotechnology Corp., Shanghai, China). Samples containing equal amounts of proteins were electrophoresed using sodium dodecyl sulphate polyacrylamide gel electrophoresis (SDS-PAGE) on 10% gels and transferred to a polyvinylidene fluoride (PVDF) membranes. After blocking with 5% BSA solutions for 2 h at room temperature, membranes were incubated using the following antibodies: LBR (diluted 1 : 500, ab32535; Abcam, Cambridge, UK), p53 (diluted 1 : 1000, ab26; Abcam), p21 (diluted 1 : 1000, ab109199; Abcam), p16 (diluted 1 : 1000, ET1602-9; HUABIO, Hangzhou, China), METTL14 (diluted 1 : 1000, 26158-1-AP; Proteintech, Wuhan, China), WTAP (diluted 1 : 1000, 60188-1-Ig; Proteintech), ALKBH5 (diluted 1 : 1000, 16837-1-AP; Proteintech), YTHDC1 (diluted 1 : 1000, A7318; ABclonal, Wuhan, China), YTHDC2 (diluted 1 : 1000, A15004; ABclonal), METTL3 (diluted 1 : 1000, db318; Diagbio, Hangzhou, China), and *β*-actin (as the loading control) (diluted 1 : 1000, 3700; CST, Danvers, USA) at 4°C overnight. They were then washed three times with TBST and then incubated with secondary antibody conjugated to horse radish peroxide (HRP) (diluted 1 : 5000, A0208; Beyotime, Shanghai, China). Specific immune complexes were exposed with ECL Western Blotting Substrate (NCM Biotech, Suzhou, China) and detected using the Syngene GeneGenius gel imaging system (Syngene, Cambridge, UK).

### 2.5. m^6^A Dot Blot Assay

Total RNA isolated from tissues or cells, as described above, was dissolved in 3 times volume of RNA incubation buffer and denatured at 65°C for 5 min. Then, the samples were divided into subgroups of 200 ng, 100 ng, or 50 ng. These were dissolved in SSC buffer (Sangon Biotech, Shanghai, China), loaded to an Amersham Hybond-N+ membrane (GE, Healthcare, USA) which was settled on the Bio-Dot Apparatus (Bio-Rad, USA). The membrane was then crosslinked by UV light for 5 min, followed by staining with 0.02% methylene blue (Sangon Biotech, China). The membranes were scanned to indicate the total content of input RNA. After blocking with 5% skimmed milk, membranes were incubated with specific m^6^A antibody (diluted 1 : 1000, #202003; Synaptic System, Germany) overnight at 4°C. Following washing, the membranes were incubated with HRP-conjugated anti-rabbit immunoglobulin G (IgG) for 2 h before being visualized using the imaging system. The results of dot blotting were obtained using Syngene GeneGenius gel imaging system (Syngene, Cambridge, UK) and quantified by ImageJ. Methylene blue (MB) staining was used as loading control.

### 2.6. RNA Decay Assay

To measure RNA stability, an RNA decay assay was performed. CCD-18Co cell line was cultured in three 6-well plates followed by the treatment of METTL14 knockdown. Actinomycin D (Selleck, S8964) was added into each well at a final concentration of 5 *μ*g/ml. Cells were collected at 0, 2, or 4 h. Total RNA was extracted, and qPCR was performed to quantify the relative abundance of *LBR* mRNA (relative to 0 h).

### 2.7. Methylated RNA Immunoprecipitation (MeRIP)

m^6^A antibody (Synaptic System; #202003) and normal rabbit IgG (Beyotime Biotechnology, #A7028) were conjugated to protein A/G mixed magnetic beads, respectively (Beyotime Biotechnology, #A7016), overnight at 4°C. Total RNA was incubated with the antibody in immunoprecipitation buffer supplemented with RNase inhibitor overnight at 4°C. RNA was then eluted from the beads and extracted, and the m^6^A-enriched RNA and the input reversely transcribed into cDNA. Enrichment was detected by qPCR. Relative enrichment of m^6^A was normalized to the input: %input = 1/2^Ct [IP]–Ct [Input]^.

### 2.8. Cell Proliferation

To determine the clonal expansion efficiency of the cells from the different treatment groups, 2000 cells were seeded in 6-well plate. About 10-15 days later, the cells were fixed with 4% paraformaldehyde for 15 min and then stained with 0.2% crystal violet staining solution (Beyotime Institute of Biotechnology Corp., Shanghai, China) for 20 min at room temperature. Crystal violet was then eluted using 10% acetic acid, and the OD was examined at 595 nm using a spectrophotometer.

### 2.9. SA-*β*-*Gal* Staining

SA-*β*-*Gal* activity was measured using a SA-*β*-*Gal* staining kit (Beyotime Institute of Biotechnology Corp., Shanghai, China). Specifically, cells were washed three times with PBS and fixed for 15 min at room temperature for 15 min in the fixing solution of the SA-*β*-*Gal* kit. After washing in PBS three times, cells were incubated in senescence detection solution overnight at 37°C. The cells were then observed under a light microscope. SA-*β*-*Gal* activity was expressed as the percentage of SA-*β*-*Gal* staining-positive cells to total cells.

### 2.10. Immunofluorescence Staining

Immunofluorescence experiments were performed as follows. CCD-18Co cells were grown on coverslips. Cells were fixed for 15 min at room temperature (RT) in a 4% paraformaldehyde solution before being permeablized by PBS with 0.1% Triton-X-100 for 10 min at RT. Coverslips were then washed with PBS and blocked with 5% BSA in PBS. Primary antibody was incubated for 2 h and secondary antibody for 1 h at room temperature. An anti-m^6^A antibody (#202003) was used at 1 *μ*g/ml for immunofluorescence. Alexa 568-conjugated anti-rabbit (diluted 1 : 200, Invitrogen, USA) was used as the secondary antibodies for immunofluorescence analysis.

### 2.11. Animals

The C57BL/6 mice used in this experiment were obtained from SLAC Laboratory Animal Co. Ltd. (Shanghai, China) and housed in a specific pathogen-free (SPF) level room. The room temperature was maintained at 22°C with 12 h light/dark cycle. Mice were provided with ad libitum water and food. All animal studies were performed in accordance with the guidelines of the Institutional Animal Use and the Animal Experimentation Ethics Committee of Zhejiang University School of Medicine (20161206-21). The following fly strains were used: w^1118^, *tubulin-Gal4*, and *UAS-Mettl14-RNAi*.

### 2.12. Fly Lifespan Assay

Flies were reared on standard cornmeal medium at 25°C. Progeny was reared at standard density under 12L:12D at 25°C and 70% humidity. Adult flies were collected in standard culture vials with a maximum of 10 flies per vial. Flies were counted daily and transferred to fresh vials every other day. The lifespan of the fly was calculated as the number of days it survived postemergence.

### 2.13. Climbing Assay

In brief, cohorts of 20 flies of the indicated age and genotype were transferred into two empty vials which were connected at their opening with adhesive tape, creating an enclosed transparent tube, approximately 15 cm tall. After the transfer, flies were allowed to rest for 30 minutes at room temperature. The tubes were then tapped to bring all the flies to the bottom. The movement of the flies during the experiments was recorded using a digital camera. The number of flies passing the 8 cm mark on the vial over 20 seconds was counted. For each cohort of 30 flies, the experiment was repeated 3 times. A total of two cohorts from separate crosses were analyzed and scored for each group.

### 2.14. Smurf Assay

Gut barrier function was analyzed by placing flies on blue food (FD&C blue dye#1, E8500 blue dye, Solarbio, Beijing, China) postfeeding [34]. Briefly, each group was transferred onto fresh medium containing blue dye (2.5% w/v) at 8 a.m. for 12 h. “Smurf” flies were defined by visible blue food dye seen throughout the body (not limited to the proboscis and crop), which suggests disruption of gut integrity. A fly was counted as “Smurf positive” when blue dye could be observed outside of the digestive tract [35].

### 2.15. Statistical Analysis

Data was expressed as mean ± standard deviation. Student's test was used to analyze the differences between two groups. A one-way ANOVA or two-way ANOVA analysis was used to compare the differences among more than two groups. A *p* value <0.05 was regarded statistically significant. Statistical analyses were performed using the GraphPad Prism 8.0 Software (GraphPad, Inc., San Diego, USA).

## 3. Results

### 3.1. The m^6^A Modification and the Expression Level of Mettl14 Were Decreased in Aged Mice and *Drosophila*

**Figure 1:**
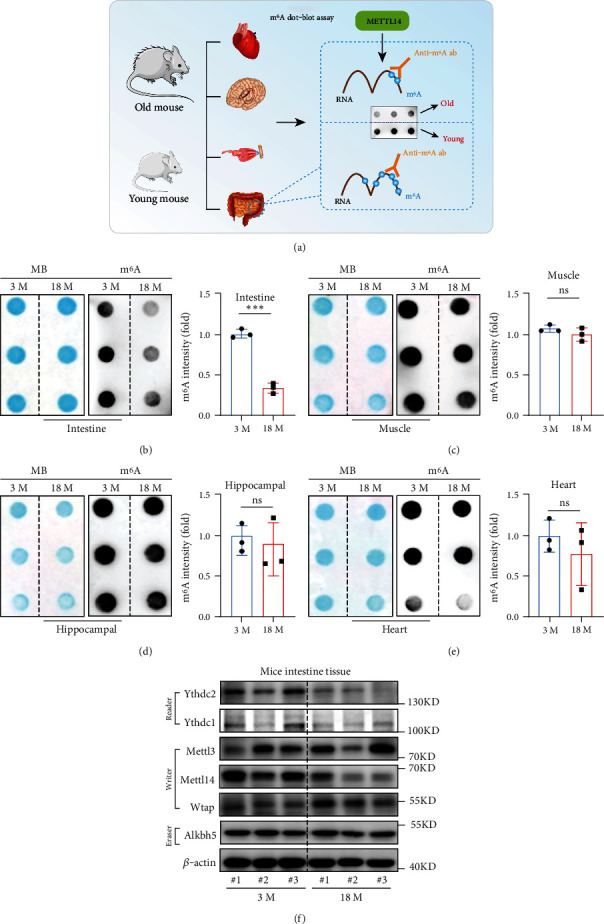
m^6^A modification and the expression of Mettl14 were downregulated in the colon along with the aging process. (a) Flow diagram of the study design. (b–e) The m^6^A modification levels in the intestine, muscle, hippocampus, and heart collected from 3-month and 18-month old mice were tested by m^6^A dot blot assays. Methylene blue staining (left) was used to detected input RNA, while the intensity of dot immunoblotting (right) represents the level of m^6^A modification. Data are expressed as mean ± SD of three independent biological experiments. Student *t*-test. ns: not significant;  ^∗∗∗^*p* < 0.001. (f) The protein expression level of the key enzyme of m^6^A modification in intestines from old (18 month) or young (3 month) mice.

To investigate whether m^6^A modification level changes with aging, we firstly performed m^6^A dot blot assay to detect m^6^A level in various tissues of 18-month or 3-month old mice (Figure 1(a)). Results showed that m^6^A level was decreased in colon tissues of 18-month-old mice as compared to that of 3-month-old mice but not in other tissues from the muscle, hippocampus, or heart (Figures 1(b)–1(e)). The decreased m^6^A level could be related to the dysregulation of the m^6^A methylase complex or demethylases [36]. Western blot analysis of key enzymes showed that the expression of Mettl14 was significantly downregulated in intestine tissues of 18-month mice, compared to that in the 3-month group (Figure 1(f)), which were consistent with the qPCR data (Figure S1A). To examine whether Mettl14 was downregulated during aging in other organisms, we analyzed the m^6^A modification levels in flies and found that the mRNA expression of *Mettl14* was significantly decreased in 40-day old flies as compared to that in 5-day old flies (Figure S1B). These observations suggest that decreased Mettl14 expression levels are associated with the aging process.

### 3.2. The Modification of m^6^A Was Downregulated in Senescent Cells

CCD-18Co, a colon fibroblast cell line [37], was used to evaluate the effect of m^6^A modification on cellular senescence. As shown in Figure S2A, the proliferation of CCD-18Co had slowed down along with the passage number, suggesting that passage 4 (P4) cells were in a relatively juvenile status, while passage 20 (P20) cells were senescent. CCD-18Co cells were treated with doxorubicin to generate drug-inducible senescence. An inhibited clone formation ability indicated the successful establishment of senescent cell model (Figure S2A). We also performed SA-*β*-*Gal* staining, one of the primary criteria for identifying senescent cells. A higher proportion of senescent cells were observed than that in control cells (Figure S2B).

**Figure 2:**
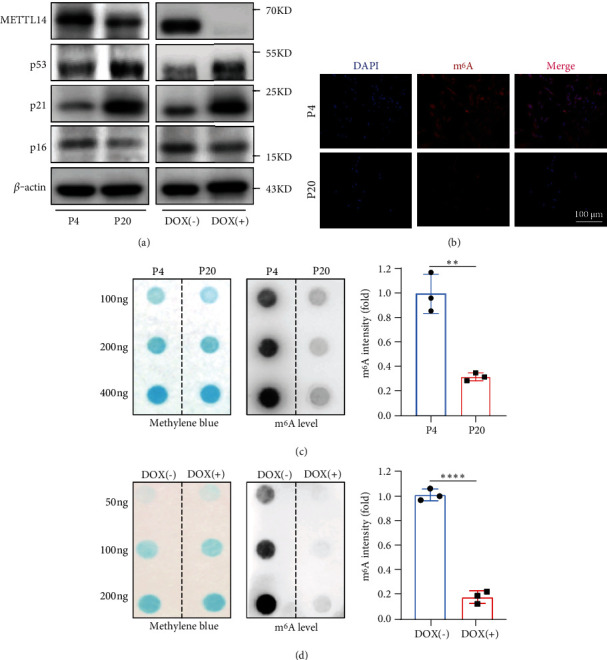
Alteration of m^6^A modification level and METTL14 expression with aging. (a) Western blot analysis of METTL14 protein levels in two cellular models. (b) Immunofluorescence analyses were conducted to evaluate the m^6^A modification levels at different passages. Scale bar, 100 *μ*m. (c) Global m^6^A level of RNA extracted from different passages cells was measured via m^6^A dot blot assays. RNAs were serially diluted and loaded equally with 100 ng, 200 ng, or 400 ng. Data are expressed as mean ± SD of three independent biological experiments. Student *t*-test.  ^∗∗^*p* < 0.01. (d) Global m^6^A level of RNA extracted from DOX-induced senescent CCD18-Co cells was measured via m^6^A dot blot assays. RNAs were serially diluted and loaded equally with 50 ng, 100 ng, or 200 ng. Methylene blue staining (left) was used to detected input RNA, while the intensity of dot immunoblotting (right) represented the level of m^6^A modification. Data are expressed as mean ± SD of three independent biological experiments. Student *t*-test.  ^∗∗∗∗^*p* < 0.0001; P4: passage 4; P20: passage 20; DOX: doxorubicin.

We then examined the expression of METTL14 in the cells, showing downregulated METTL14 protein levels in both the replicative and inducible senescence cell models. The expression level of p53 and p21, which were markers of senescence, was negatively correlated with that of METTL14 (Figure 2(a)). An m^6^A modification level was also analyzed using immunofluorescence. As expected, the m^6^A level was reduced in cells of a higher passage number (Figure 2(b)). We further detected m^6^A methylation by m^6^A mRNA dot blot and found that the overall m^6^A level declined both with increasing passages and upon DOX-induced cellular senescence (Figures 2(c) and 2(d)).

### 3.3. METTL14 Regulates m^6^A Modification-Dependent Colon Fibroblast Senescence

**Figure 3:**
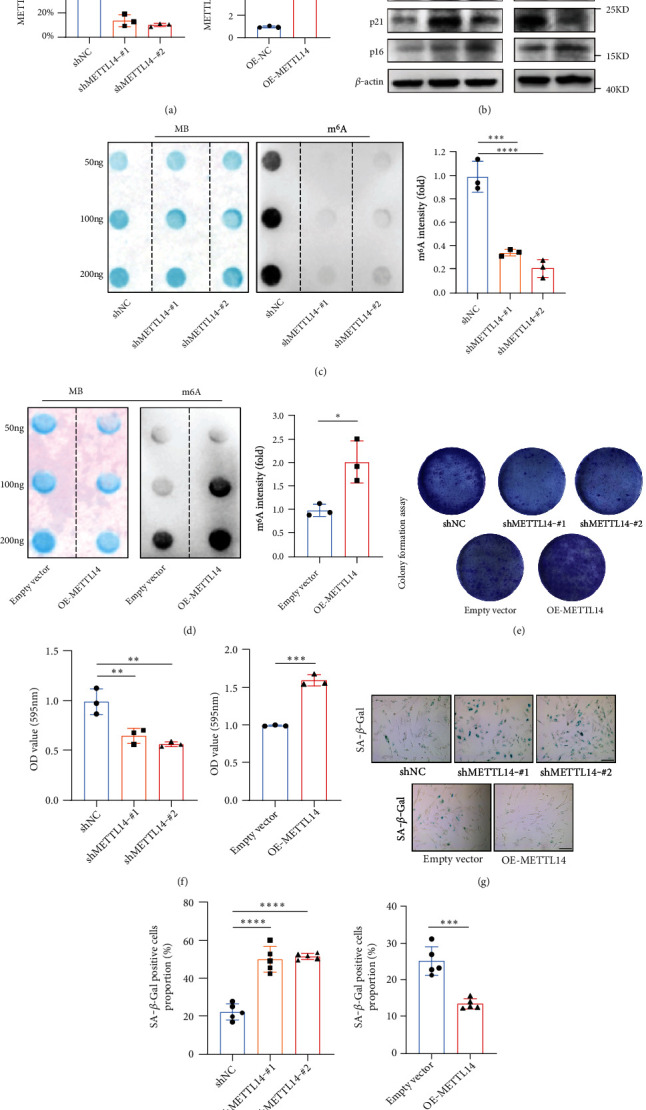
Functional analysis of the METTL14 role in cellular senescence. (a, b) The mRNA and protein expression levels of METTL14 were measured by RT-qPCR and western blotting, respectively, after transfection with the shRNA targeting METTL14 plasmid and pLV-METTL14 plasmid. *β*-Actin was used as the internal control. Data are expressed as mean ± SD of three independent biological experiments. Student *t*-test.  ^∗∗∗∗^*p* < 0.0001. (c, d) Global m^6^A level of RNA extracted from METTL14-silenced or METTL14-overexpressed CCD18-Co cells was measured using dot blot assays. RNAs were loaded equally to 50 ng, 100 ng, or 200 ng. Data are expressed as mean ± SD of three independent biological experiments. One-way ANOVA followed by Tukey's test (c). Student *t*-test (d).  ^∗∗∗^*p* < 0.001;  ^∗^*p* < 0.05. (e, f) Colony formation assay in METTL14-silenced or METTL14-overexpressed CCD18-Co cells. The stained cells were eluted using 10% acetic acid, and the absorbance of extracted strain was measured at 595 nm to quantify. Data were presented as mean ± SD, *n* = 6. One-way ANOVA followed by Tukey's test (e). Student *t*-test (f).  ^∗∗^*p* < 0.01;  ^∗∗∗^*p* < 0.001. (g, h) Analysis of SA-*β-Gal* activity in METTL14-silenced or METTL14-overexpressed CCD18-Co cells. Scale bar, 200 *μ*m. Data were presented as mean ± SD, *n* = 5. One-way ANOVA followed by Tukey's test (g). Student *t*-test (h).  ^∗∗∗^*p* < 0.001;  ^∗∗∗∗^*p* < 0.0001.

We generated METTL14-silenced or METTL14-overexpressed cell lines using short hairpin RNA (shRNA) or overexpression plasmids (Figures 3(a) and 3(b)), respectively. shRNA-mediated METTL14 knockdown led to a significant upregulation of p53 and p21 in CCD18-Co cells, while the METTL14 overexpression considerably reduced the expressions of p53 and p21 (Figures 3(a) and 3(b)). We then applied dot blot assays to determine the effects of METTL14 on m^6^A methylation level. Results showed that both METTL14 knockdown and overexpression significantly affected the m^6^A methylation level (Figures 3(c) and 3(d)), implying that METTL14 was critical for m^6^A modification. In addition, METTL14-silenced CCD-18Co cells acquired characteristics of premature ageing, as shown by decreased proliferative capacity and increased percentage of SA-*β*-*Gal-*positive cells. These results indicated that whilst METTL14 knockdown accelerated CCD-18Co senescence, its overexpression enhanced cell proliferation capacity and reduced the numbers of SA-*β*-*Gal-*positive cells (Figures 3(e)–3(h)). Collectively, METTL14 was confirmed to play a vital role in the m^6^A modification-dependent colon fibroblast senescence.

### 3.4. Knockdown of Mettl14 Led to Shorter Lifespan and Disrupted Intestinal Barrier in *Drosophila*

**Figure 4:**
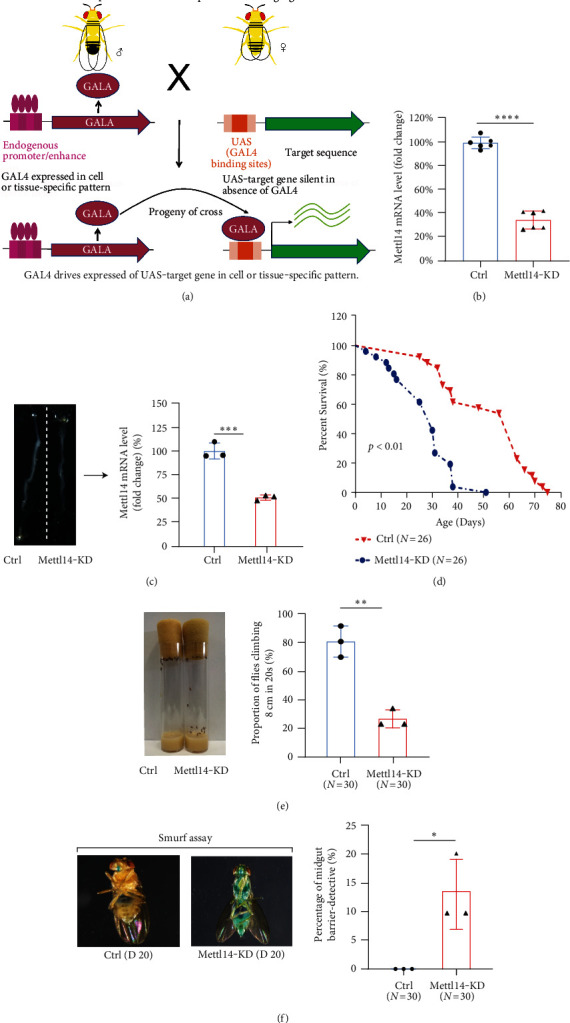
Knocking down Mettl14 shortens lifespan and increases intestinal permeability. (a) *UAS-Mettl14 RNAi* and *tubulin-GAL4* flies were intercrossed to generate *UAS-Mettl14 RNAi* and *tubulin-GAL4* flies. In the *GAL4-UAS* system, the transcription activator protein GAL4 binds to the upstream activation sequence (*UAS*) and activates *Mettl14 RNAi* transcription to knock down Mettl14 in *Drosophila melanogaster*. (b) The knockdown efficiency of *Mettl14* in transgenic flies was tested by RT-qPCR. Data are expressed as mean ± SD of three independent biological experiments. Student *t*-test.  ^∗∗∗∗^*p* < 0.0001. (c) Verification of intestine dissected from transgenic flies *Mettl14* knockdown by RT-qPCR. left is a dissection showing the *Drosophila* intestinal tract. Data are expressed as mean ± SD of three independent biological experiments. Student *t*-test.  ^∗∗∗^*p* < 0.001. (d) Survival curves of control and transgenic flies. The log-rank (Kaplan-Meier) test was performed. (e) Comparison of climbing ability of control and transgenic flies at day 20. Data are expressed as mean ± SD of three independent biological experiments. Student *t*-test.  ^∗∗^*p* < 0.01. (f) Smurf gut permeability assay in 20-day old control and *Mettl14 RNAi* flies. Data are expressed as mean ± SD of three independent biological experiments. Student *t*-test.  ^∗^*p* < 0.05.

To investigate the role of METTL14-mediated m^6^A modification in aging, we performed Mettl14 knockdown experiments in *Drosophila* (Figure 4(a)). The silencing efficiency of *Mettl14* was validated by RT-qPCR using the whole fly sample (Figure 4(b)). We dissected gastrointestinal tracts of the flies and examined *Mettl14* expression levels. A significantly downregulated level was noted in the intestine tissues of *Mettl14* knockdown flies, as compared with that in controls (Figure 4(c)).

In *Drosophila*, climbing ability was measured to evaluate locomotor behavior, so we evaluated the lifespan together with climbing ability of the flies (Figure S3). Results showed that Mettl14 knockdown flies had a short lifespan (Figure 4(d)) and exhibited a weaker creep ability than control flies (Figure 4(e)). This correlated with the suggestion that the loss of intestinal integrity could be used as an organismal aging marker for flies [38]. We performed Smurf assay in Mettl14 knockdown flies and observed that knockdown of Mettl14 disrupted intestinal integrity, with significantly increased Smurf-positive flies, compared to the controls (Figure 4(f)). These results suggested that Mettl14 played an essential role in regulating locomotor behavior, intestinal integrity, and aging in *Drosophila*.

### 3.5. *LBR* Was Identified as an m^6^A-Targeted Gene

**Figure 5:**
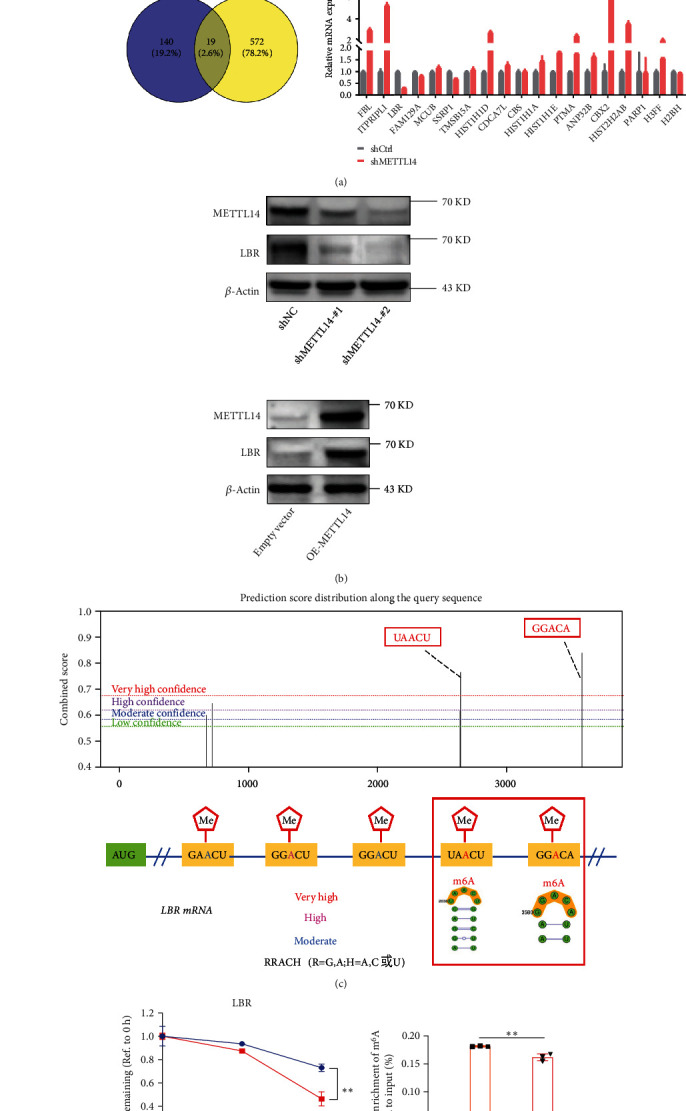
*LBR* is a key downstream gene of METTL14. (a) Schematic diagram showing the screening criterion for METTL14 targets. Venn diagram of all the possible overlapping transcripts in nine comparison groups sequenced together, showing reduced abundance with senescence and filtering for a significant FDR < 0.15. RNA-seq data was acquired from GEO datasets (GSE130727). RT-qPCR was performed in CCD18-Co with METTL14 knockdown to validate the overlapped genes. The *LBR* was consistent among 19 genes. *FBL*: fibrillarin; *ITPRIP*: inositol 1,4,5-trisphosphate receptor interacting protein; *LBR*: lamin B receptor; *FAM129A*: family with sequence similarity 129 member A; *MCUB:* mitochondrial calcium uniporter dominant negative subunit beta; SSRP1: structure specific recognition protein 1; *TMSB15A*: thymosin beta 15A; *HIST1H1D*: H1.3 linker histone, cluster member; *CDCA7L:* cell division cycle associated 7 like; *CBS*: cystathionine beta-synthase; *HIST1H1A:* H1.1 linker histone, cluster member; *HIST1H1E*: H1.4 linker histone, cluster member; *PTMA*: prothymosin alpha; *ANP32B*: acidic nuclear phosphoprotein 32 family member B; *CBX2*: chromobox 2; *HIST2H2AB:* histone cluster 2 H2A family member B; *PARP1*: poly(ADP-ribose) polymerase 1; *H3FF*: H3 clustered histone 11; *H2BH*: H2B clustered histone 9. (b) The expression of LBR following METTL14 knockdown or overexpression was evaluated by western blotting. (c) Prediction of the specific m^6^A methylation loci of *LBR* using the SRAMP website. (d) METTL14-silenced cells were treated with actinomycin D and harvested at 0, 2, or 4 h. RNA decay rates were determined to estimate the stability of *LBR* mRNA (normalized to the expression at 0 h). Data are expressed as mean ± SD of three independent biological experiments. Two-way ANOVA analysis.  ^∗∗^*p* < 0.01. (e) m^6^A modification of *LBR* was detected by MeRIP-qPCR analysis using anti-IgG and anti-m^6^A antibodies. Relative m^6^A enrichment of *LBR* mRNA for each IP group was normalized to input. Data are expressed as mean ± SD of three independent biological experiments. One-way ANOVA followed by Tukey's test.  ^∗∗^*p* < 0.01.

The Myriam Gorospe group, aimed to identify robust shared markers of senescence, performed RNA-sequencing analysis across 8 diverse models of senescence triggered in human diploid fibroblasts (WI-38, IMR-90) and endothelial cells (HUVEC, HAEC) by replicative exhaustion, exposure to ionizing radiation or doxorubicin, and expression of the oncogene HRAS-G12V [39]. On the basis of the data (GSE130727) presented above, the same data was reanalyzed. Considering that there was a positive correlation between m^6^A modification levels and mRNA expression levels [20], we focused on protein-coding genes that were downregulated in nine comparison groups. Using a Venn diagram, a total of 19 common candidate key genes were unveiled. We then conducted RT-qPCR to evaluate the impact of METTL14 on each candidate gene. Among these, LBR was significantly downregulated following METTL14 silencing (Figure 5(a)). We further confirmed that METTL14 knockdown resulted in decreasing LBR protein level in CCD18-Co cells and the overexpression of the METTL14 enhanced LBR expression (Figure 5(b)). These data indicated that *LBR* could be a target gene of METTL14.

To identify the specific m^6^A methylation loci of *LBR*, we interrogated the *LBR* sequence using the SRAMP website (http://www.cuilab.cn/sramp/) [40] and screened out 2 positions with a likely abundance of m^6^A methylation loci (Figure 5(c)). We also found that METTL14 knockdown induced a quicker degradation rate of *LBR* mRNA (Figure 5(d)). Therefore, we conducted a MeRIP-qPCR assay to determine the enrichment of m^6^A in *LBR*. Compared with IgG control, the m^6^A-specific antibody had robustly enriched *LBR* transcripts. There was a decreased amount of *LBR* modified by m^6^A following METTL14 knockdown (Figure 5(e)). Collectively, these results indicate that *LBR* could be a potential target gene of METTL14 in the m^6^A modification-dependent way.

### 3.6. METTL14 Delayed the Cellular Senescence through the Regulation of LBR

**Figure 6:**
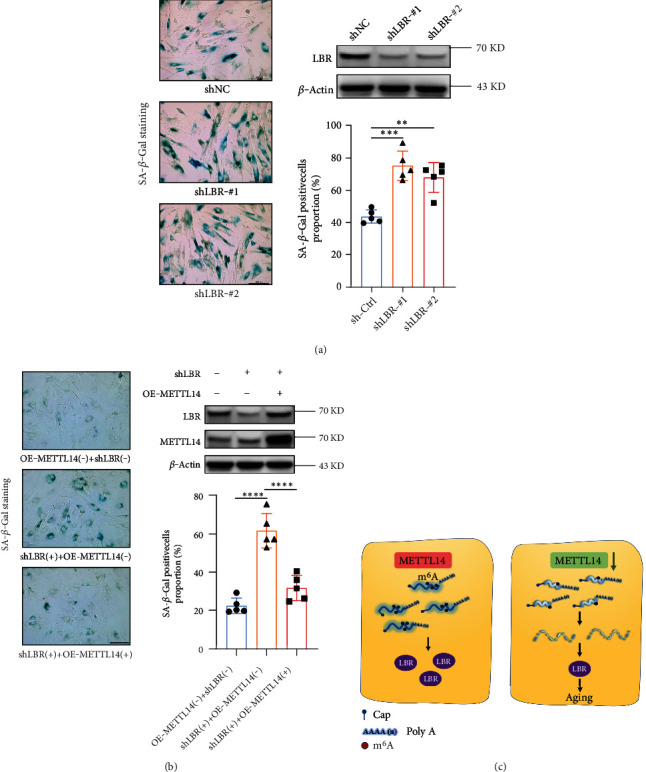
METTL14 delayed aging via stabilizing *LBR* mRNA. (a) Induction of cellular senescence in CCD18-Co cells by knocking down LBR. Western blotting was applied to detect the transfection efficiency of METTL14 shRNA. *β*-Actin was used as the internal control. The percentage of SA-*β*-*Gal*-positive cells was manually counted. Scale bar, 200 *μ*m. Data were presented as mean ± SD, *n* = 5, biologically independent experiments. One-way ANOVA followed by Tukey's test.  ^∗∗^*p* < 0.01;  ^∗∗∗^*p* < 0.001. (b) The efficiency of the cotransfection of METTL14 overexpression plasmid and *LBR* shRNA into CCD18-Co cells was verified by western blotting. Rescue experiments were conducted to determine the influence of LBR knockdown on METTL14 overexpression cells, with bar charts showing the percentage of SA-*β*-*Gal*-positive cells. Data were presented as mean ± SD, *n* = 5, biologically independent experiments. One-way ANOVA followed by Tukey's test.  ^∗∗∗∗^*p* < 0.0001. (c) A schematic illustration to summarize our findings relating to METTL14-guided m^6^A modulation on LBR. METTL14 could maintain m^6^A levels to stabilize *LBR* mRNA. Contrastingly, METTL14 downregulation resulted in reduced *LBR* m^6^A levels, together with cellular senescence.

To uncover the role of LBR in regulating cell senescence, we performed LBR-specific shRNA knockdown in CCD-18Co cells. The SA-*β*-*Gal* staining showed that LBR knockdown clearly increased the ratios of blue-stained cells compared with those of the controls (Figure 6(a)). To investigate whether this phenotype was mediated by a dysregulation of the METTL14-LBR, CCD-18Co cells were cotransfected with an METTL14 overexpression plasmid and a shRNA plasmid targeting LBR. Western blotting analysis consistently verified the transfection efficiency. The SA-*β*-*Gal* staining assays showed that the ratios of blue-stained cells increased by LBR knocking down was reversed by transfection with METTL14 plasmid (Figure 6(b)).

## 4. Discussion

Over recent years, most developed countries have become increasingly prone to an aging population [41]. This may have paved the way for an increasing proportion of research focusing on molecular mechanisms associated with aging. An increase in intestinal permeability would allow bacterial components (e.g., LPS) to enter the bloodstream [42]. Growing evidence indicated that chronic inflammation accelerates senescence, while inflammation deletion delays senescence [43]. To our knowledge, disruption of the epithelial integrity allowed for enhanced permeability, bacterial translocation, and inflammation leading to inflammaging.

Growing evidence showed that m^6^A modification had an essential effect on some underlying diseases including cancers [42] and participated in multiple physiological and pathological processes by modulating different target pathways. m^6^A modification was essential for the maintenance of colonic epithelial homeostasis, and mice with colon-specific KO of Mettl14 showed disturbed integrity of the colonic epithelial barrier [43]. Wang et al. recently uncovered a novel antiviral function of m^6^A modification during rotavirus (RV) infection in small bowel intestinal epithelial cells (IECs) [44]. Similar study supported a model in which m^6^A modifications in the *Traf6* transcript regulated the intestinal immune response to bacterial infection via a complex consisting of *YTHDF1*, *DDX60*, and the *Traf6* transcript [45].

Epigenetic modifications have been suggested to play central roles in cell differentiation and senescence [46]. Lence et al. demonstrated that m^6^A modulates neuronal functions and sex determination in *Drosophila*, revealing that loss of function of the methyltransferases produced severe behavioral defects [47]. METTL3, in particular, was seen to be reduced in prematurely senescent hMSC models. Conversely, METTL3 was recognized as active in the alleviation of hMSC senescence through m^6^A modification-dependent stabilization of the *MIS12* transcript [20].

We examined the m^6^A modification levels in a variety of mouse organs associated with different age groups. We found that the m^6^A modification level exhibited a particular decline in colon tissues of old mice. Zhang et al. had previously uncovered a unique m^6^A-regulation-expression pattern in the human brain and intestine tissues and confirmed m^6^A modification and enrichment to be organ-specific processes [48]. Similarly, as m^6^A regulators, the altered expression of METTL3 and METTL14 has been observed in various diseases and age-related processes where METTL3/14 was observed as progressively and notably declined in human F2S fibroblasts undergoing replicative senescence [19]. METTL14 is also a noted participator in the regulation of T cell homeostasis [49], and its knockdown in embryonic mouse brains disturbed embryonic cortical neurogenesis [50].

In our study, METTL14 was identified to regulate m^6^A modification levels linked to senescence in animal models and in colon fibroblast cells. Our study confirmed *LBR* as a target gene of METTL14 and showed that the expression of LBR was altered via m^6^A modification in the process of cellular senescence. Specific RNA modification sites are essential for m^6^A modification [51]. m^6^A modification clearly exerts its biological function via interplay with binding proteins [52] to directly or indirectly affect RNA function [53]. We uncovered that the m^6^A modification might promote the mRNA stability of *LBR* via the regulation of METTL14. However, the specific modification sites in *LBR* and further validation was warranted. Interestingly, these reader proteins had very different dynamics among the different species. Ythdc1 genes showed different expression levels in different species. The gene expression of Ythdc1 was downregulated in *Drosophila* melanogaster while upregulated in mice intestine during aging process in RNA levels (Figure S1). Whether this process has any interplay with other m^6^A reader proteins such as IGF2BP1/2/3 [54–56] and YTHDF1 [18] requires further study.

In summary, our findings demonstrate that METTL14-mediated m^6^A modification can delay cellular senescence, potentially through enhancing the stability of *LBR* mRNA (Figure 6(c)). Our *Drosophila* model with Mettl14 depletion showed a shorter lifespan and disrupted intestinal integrity. Overall, our results might suggest a potential target for antiaging therapies.

## Figures and Tables

**Table 1:** Sequences of shRNAs (5′-3′).

sh-NC	GGTTCTCCGAACGTGTCACGT
sh-*METTL14*#1	CCATGTACTTACAAGCCGATA
sh-*METTL14*#2	GCCGTGTTAAATAGCAAAGAT
sh-*LBR*#1	TGATTGGATGGGTGGTTATTA
sh-*LBR*#2	TTCCCACCAGGCCGACATTAA

**Table 2:** Information of the RT-qPCR primers.

Primers	Sequencing (5′-3′)
Mouse *β-actin*-F	TGGCTCCTAGCACCATGAAG
Mouse *β-actin*-R	CGCAGCTCAGTAACAGTCCG
Mouse *Mettl14*-F	CTTCGACCGAAGTCACCTCC
Mouse *Mettl14*-R	CTACCGAGGAGTAAAGCCGC
Mouse *Mettl3*-F	GATAGTCCCGTGCCTACTGC
Mouse *Mettl3*-R	TGGCGTAGAGATGGCAAGAC
Mouse *Wtap*-F	CATTTTGTGGCTGCGAGACC
Mouse *Wtap*-R	TCTGTTTCACTCAGTCGGACC
Mouse *Alkbh5*-F	CTTTGCTTCGGCTGCAAGTT
Mouse *Alkbh5*-R	AATGTCCTGAGGCCGTATGC
Mouse *Ythdc1*-F	CGTAGGAAGCTGAGTGGAGC
Mouse *Ythdc1*-R	TCCCCATCTTTCTCCTCCCG
Mouse *Ythdc2*-F	GCTCATGCAATGATGACCTGT
Mouse *Ythdc2*-R	AATGCCATTGTTGAGTCGCC
Drosophila *RP49*-F	TGGTTTCCGGCAAGCTTCAA
Drosophila *RP49*-R	TGTTGTCGATACCCTTGGGC
Drosophila *Mettl14*-F	AGGAAGAGGAGTTTGGCAGC
Drosophila *Mettl14*-R	GCTGGAGCCTCAGCAAAGTA
Drosophila *Mettl4*-F	TACTGCACCAACTTCTGCCC
Drosophila *Mettl4*-R	AGCTTCGAGAGCGGTATGTG
Drosophila *Mettl3*-F	GCACAAAAGTCGAGTGCCTG
Drosophila *Mettl3*-R	CTGCCGATTCTGTCTTGGGT
Drosophila *fl(2)d*-F	GTGGTATTGGAGGAGTGTATGC
Drosophila *fl(2)d*-R	CGATTGCTTCGTTATCTCTGGC
Drosophila *Ythdc1*-F	GCGAAAAGTAGCGAGAGAAAGC
Drosophila *Ythdc1*-R	GGTTTGGCACACACGATACA
Human *β-actin*-F	AGAGCTACGAGCTGCCTGAC
Human *β-actin*-R	AGCACTGTGTTGGCGTACAG
Human *FBL*-F	GCCCACACCTTCCTGCGTAATG
Human *FBL*-R	TGCGGCTTCATGTTCTCCTGTTG
Human *ITPRIPL1*-F	TGAGCAGAGGCAGAAGGCAGAG
Human *ITPRIPL1*-R	GTTCCACAGGTTTCCCAGCATCC
Human *LBR*-F	GGAAGTTAAATTGACTCCGCTG
Human *LBR*-R	AGGTGCGTCATTTCTCTCAATA
Human *FAM129A*-F	GCAACAGAGGACACAGCAGGAC
Human *FAM129A*-R	CAAAGAACCCGAGGCAGTGATGG
Human *MCUB*-F	GGATATCATGGAGCCAGTTACA
Human *MCUB*-R	TCAGGGATTCTTTAGCCTTAGC
Human *SSRP1*-F	GTCTGTTTTTGTTACCCCACAA
Human *SSRP1*-R	TTCTTCCTCGTTCATGTTCAGA
Human *TMSB15A*-F	GCCAGACTTGTCGGAAGTGG
Human *TMSB15A*-R	TGCTGGATAGTTTCCTTTGAGGGA
Human *HIST1H1D*-F	TGTGAAGAAAAAGGCGAAGAAG
Human *HIST1H1D*-R	GCCTTGGTGATAAGCTCAGATA
Human *CDCA7L*-F	GTCAGATTTGAGTGATGATGGC
Human *CDCA7L*-R	TTTTCTCATTCTGTAAGCGTGC
Human *CBS*-F	ACCATCGAGATCCTCCGGGA
Human *CBS*-R	GGGACGAGAGCATGTTCCCA
Human *HIST1H1A*-F	GAAGGCAAAGAAACCTGCTAAG
Human *HIST1H1A*-R	CTCTTAATGCCCAGCTTAATGC
Human *HIST1H1E*-F	CTCATTACTAAAGCTGTTGCCG
Human *HIST1H1E*-R	TGTTGTTCTTCTCCACGTCATA
Human *PTMA*-F	AGGTGATGGTGAGGAAGAGGATGG
Human *PTMA*-R	CTCGTCGGTCTTCTGCTTCTTGG
Human *ANP32B*-F	GGCTTAACAGCTGAATTTGTGA
Human *ANP32B*-R	TTGGGGAGATTTGAAACTGAGA
Human *CBX2-*F	GACTTAGATGCTAAGAGGGGTC
Human *CBX2*-R	CTTCTTCCGGATGGGATCCTTC
Human *HIST2H2AB-*F	GCCATCTGCAACTAGCCGTGAG
Human *HIST2H2AB*-R	TTGTTCTTGCCAGGCTTGTGACTC
Human *PARP1-*F	CGGAGTCTTCGGATAAGCTCT
Human *PARP1*-R	TTTCCATCAAACATGGGCGAC
Human *H3FF-*F	GGATACCAACCTGTGCGCCATT
Human *H3FF*-R	GGACAGACTTCTTGGGCTGATAG
Human *H2BH-*F	CAAGGTGCTGAAGCAAGTCCAC
Human *H2BH*-R	TGGAGGTGATGGTCGAACGCTT

## Data Availability

All data generated or analyzed during this study are available from the corresponding authors on reasonable request.

## Supplementary Materials

Supplementary Materials Figure S1: verification of Mettl14 alteration in the aging process. (A) The mRNA expression level of the key enzyme of m^6^A modification in mice intestines from different ages. Data are expressed as mean ± SD of three independent biological experiments. Student *t*-test.  ^∗^*p* < 0.05;  ^∗∗^*p* < 0.01. (B) The relative mRNA expression of m^6^A RNA modification regulators in *Drosophila* at day 5 and day 40. Data are expressed as mean ± SD of three independent biological experiments. Student *t*-test.  ^∗∗^*p* < 0.01;  ^∗∗∗∗^*p* < 0.0001. Figure S2: senescence-related phenotype. (A) Clonal formation assay of replicated and DOX-induced CCD18-Co. Data are expressed as mean ± SD of three independent biological experiments. Student *t*-test.  ^∗∗∗∗^*p* < 0.0001; P4: passage 4; P20: passage 20. (B) Representative images of SA-*β-Gal* staining of replicated and DOX-induced CCD18-Co. Scale bar, 200 *μ*m. Data are presented as means ± SD, *n* = 5. Student *t*-test.  ^∗∗∗^*p* < 0.001;  ^∗∗∗∗^*p* < 0.001; P4: passage 4; P20: passage 20; DOX: doxorubicin.
