# Supplementary material for: METTL14 Regulates Intestine Cellular Senescence through m6A Modification of Lamin B Receptor
Source: Oxid Med Cell Longev. 2022 Dec 19;2022:9096436. doi: 10.1155/2022/9096436 (PMC9792243; doi:10.1155/2022/9096436)
Supplement: Supplementary Materials — Figure S1: verification of Mettl14 alteration in the aging process. (A) The mRNA expression level of the key enzyme of m6A modification in mice intestines from different ages. Data are expressed as mean ± SD of three independent biological experiments. Student t-test. ∗p < 0.05; ∗∗p < 0.01. (B) The relative mRNA expression of m6A RNA modification regulators in Drosophila at day 5 and day 40. Data are expressed as mean ± SD of three independent biological experiments. Student t-test. ∗∗p < 0.01; ∗∗∗∗p < 0.0001. Figure S2: senescence-related phenotype. (A) Clonal formation assay of replicated and DOX-induced CCD18-Co. Data are expressed as mean ± SD of three independent biological experiments. Student t-test. ∗∗∗∗p < 0.0001; P4: passage 4; P20: passage 20. (B) Representative images of SA-β-Gal staining of replicated and DOX-induced CCD18-Co. Scale bar, 200 μm. Data are presented as means ± SD, n = 5. Student t-test. ∗∗∗p < 0.001; ∗∗∗∗p < 0.001; P4: passage 4; P20: passage 20; DOX: doxorubicin. [file 9096436.f1.docx]

**Supplementary figure legends**

**
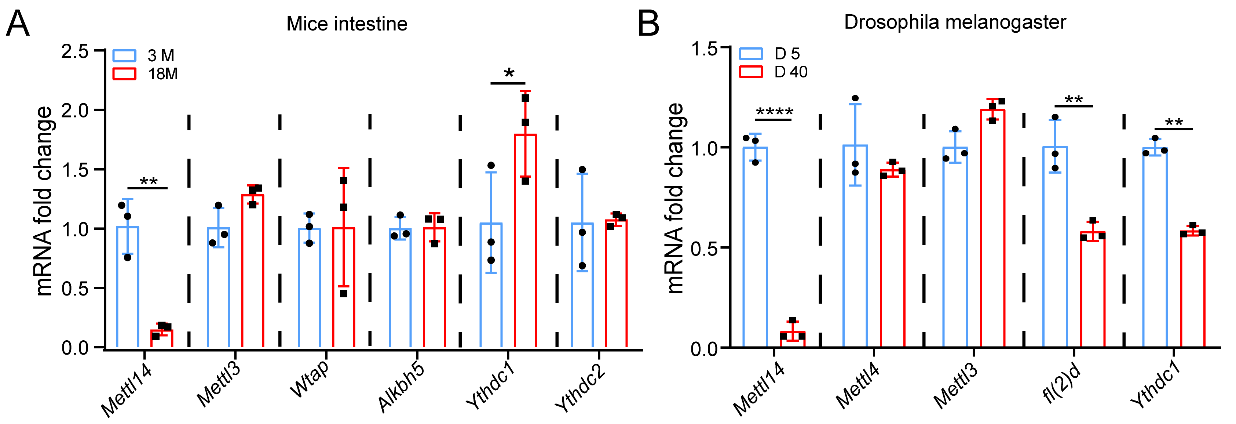
**

**Fig. S1. Verification of Mettl14 alteration in the aging process**

**A** The mRNA expression level of the key enzyme of m^6^A modification in mice intestines from different ages. Data are expressed as mean ± SD of three independent biological experiments. Student t-test. **p* < 0.05; ***p* < 0.01.

**B** Relative mRNA expression of m^6^A RNA modification regulators in *Drosophila* at day 5 and day 40. Data are expressed as mean ± SD of three independent biological experiments. Student t-test. ***p* < 0.01; *****p* < 0.0001.


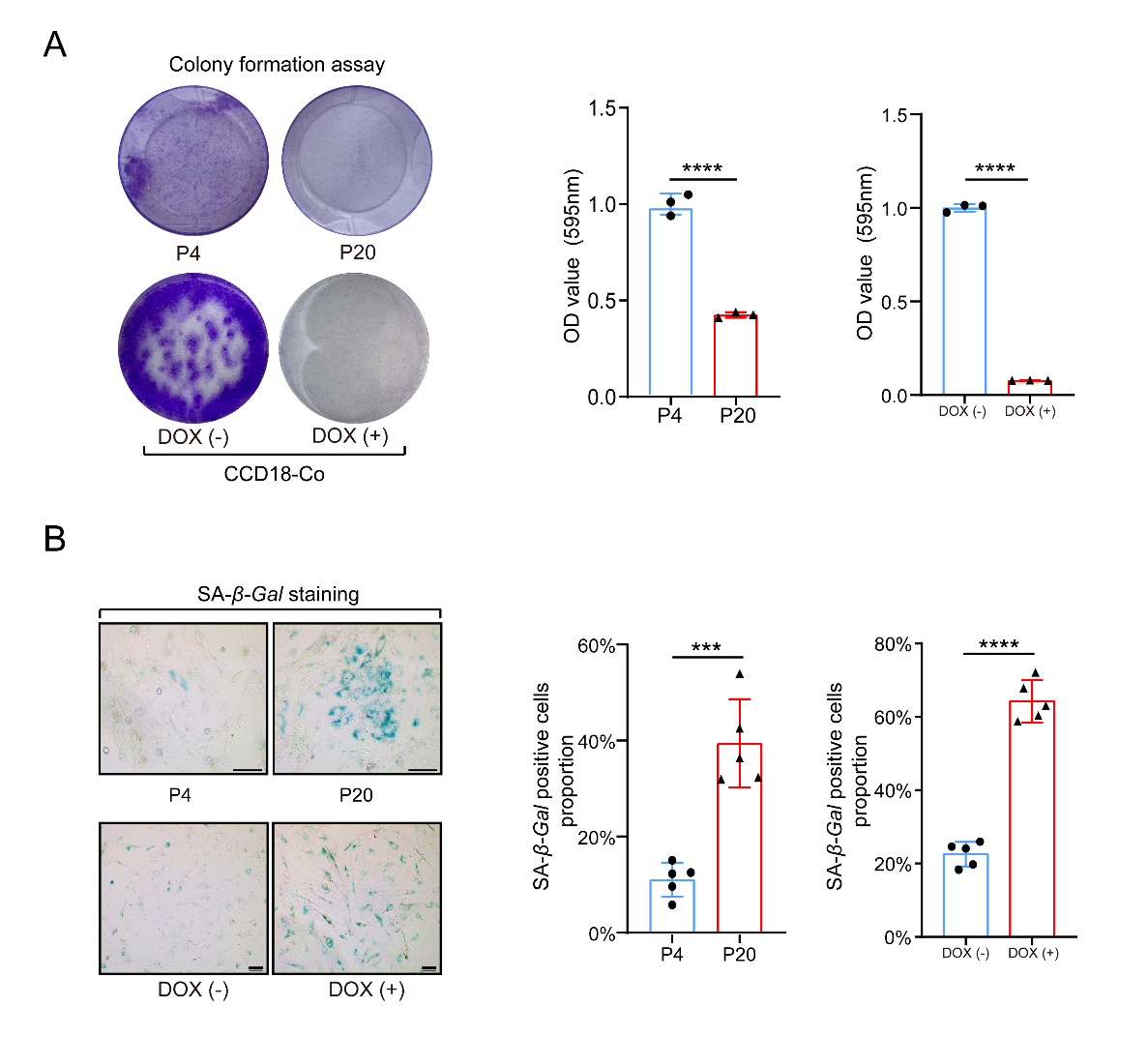


**Fig. S2. Senescence-Related Phenotype**

**A** Clonal formation assay of replicated and DOX-induced CCD18-Co. Data are expressed as mean ± SD of three independent biological experiments. Student t-test. *****p* < 0.0001; P4, passage 4; P20, passage 20.

**B** Representative images of SA-*β-Gal* staining of replicated and DOX-induced CCD18-Co. Scale bar, 200 µm. Data are presented as means ± SD, n = 5. Student t-test. ****P* < 0.001; *****P* < 0.001; P4, passage 4; P20, passage 20; DOX: doxorubicin.


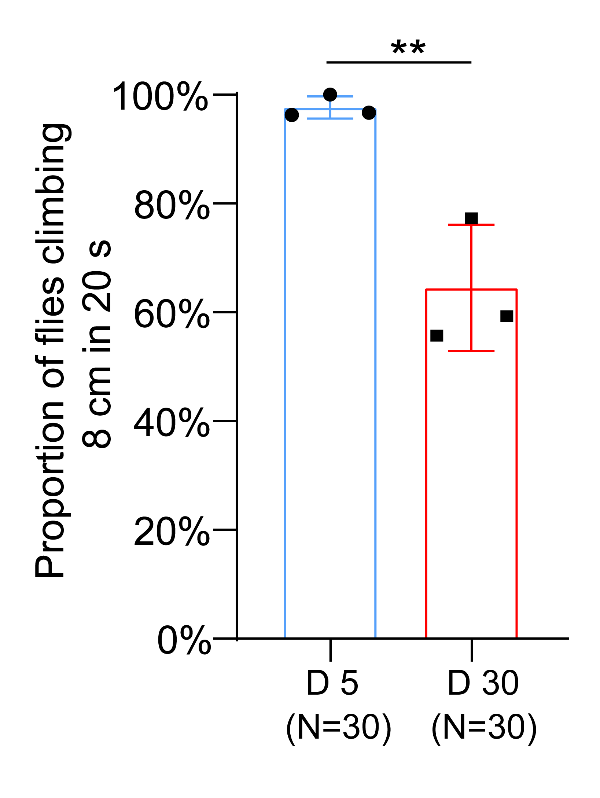


**Fig. S3. Senescence-Related Phenotype.** Comparison of climbing ability of wild-type *Drosophila* at day 5 and day 30. Data are expressed as mean ± SD of three independent biological experiments. Student t-test. ***p* < 0.01.
